# Quantitative Assessment of Masticatory Function in Patients with Temporomandibular Joint Arthralgia: A Pilot Clinical Study

**DOI:** 10.3390/jcm15072517

**Published:** 2026-03-25

**Authors:** Vinzenz Vogt, Leon Dahlmeier, Vera Colombo, Moody Kaldas, Mutlu Özcan, Aleksandra Zumbrunn Wojczyńska

**Affiliations:** Clinic of Masticatory Disorders and Dental Biomaterials, Center for Dental Medicine, University of Zurich, CH-8032 Zurich, Switzerland; vinzenz.vogt@uzh.ch (V.V.); leon.dahlmeier@gmx.ch (L.D.); moody.kaldas@zzm.uzh.ch (M.K.); mutlu.ozcan@zzm.uzh.ch (M.Ö.); aleksandra.zumbrunn@zzm.uzh.ch (A.Z.W.)

**Keywords:** clinical research, temporomandibular joint disorders, arthralgia, chewing efficiency, bite force, pressure pain threshold

## Abstract

**Objectives**: To quantitatively assess masticatory function with instrumental measures in a group of patients suffering from temporomandibular joint (TMJ) arthralgia, and to compare the results with symptom-free controls. **Methods**: Data of bite force, variance-of-hue-based (VOH) chewing efficiency, chewing frequency, the bilateral pressure pain threshold (PPT) of the temporalis and masseter muscles, and mandibular range of motion (RoM) were collected in a sample of TMJ arthralgia patients (n = 14) and controls (n = 19). The diagnosis of arthralgia was obtained following the DC/TMD protocol. Comparison between the groups was conducted using independent samples t-tests (level of significance α = 0.05). Associations within the arthralgia group were assessed using Pearson’s correlation coefficient. **Results**: In comparison to the controls, arthralgia patients showed significantly restricted pain-free and maximum unassisted mouth opening (*p* < 0.001, *p* = 0.022 respectively) as well as a significant decrease in both bite force (*p* < 0.001) and chewing frequency (*p* = 0.01). The average chewing efficiency for the arthralgia group was 0.14 ± 0.08 VOH. The PPT for both masseter muscles did not show significant differences in comparison to the control group. **Conclusions**: In patients with TMJ arthralgia, functional markers such as RoM, bite force, and chewing frequency exhibited significant limitations compared to the control group. The employment of instrumental measurements in the documentation of symptoms in clinical practice provides an objective basis for the assessment of functional limitations. Hence, we recommend integrating them into the longitudinal patients’ observation during therapy.

## 1. Introduction

Temporomandibular disorders are a prevalent condition affecting between 29.5% and 34% of the general population, with higher rates in South America compared to Asia and Europe, as well as among females and younger individuals [1,2], and an estimated increase to 44% of the general population by 2050 [3].

Involvement of the temporomandibular joint (TMJ), including disc displacement, inflammatory disorders, intra-articular loose bodies, joint trauma, and degenerative conditions are observed [4,5] and may result in TMJ arthralgia in around 17% of cases, according to the recent literature [2].

The most commonly observed symptoms in affected individuals include preauricular pain, movement restriction, and crepitation. Furthermore, TMJ dysfunction may also occur in the context of systemic polyarthritis. However, the signs of inflammation, such as redness and swelling, may manifest late in the disease process due to anatomical reasons. Changes in the TMJ, such as joint effusions or erosions, have been observed to result in unilateral jaw deviations or, in cases of bilateral involvement, an anterior open bite may arise [6]. TMJ arthralgia has the potential to impair several oral functions, including mastication, biting, yawning, speech, and laughter. This poses the risk to negatively impact patients’ quality of life. In recent years, considerable effort has been invested by experts in the field of orofacial pain to identify suitable criteria and instruments for the diagnosis of TMJ arthralgia.

Presently, the standardised examination protocol according to the Diagnostic Criteria for Temporomandibular Disorders (DC/TMDs) represents the gold standard for both research and clinical practice [7]. Nevertheless, it is not suitable for adequately capturing the functional limitations that patients frequently report experiencing in their daily lives. Furthermore, it does not involve quantitative assessments capable of monitoring symptoms that are gradually changing over time.

Therefore, the development of additional objective instruments is essential in the clinical setting for a stratified documentation of the disease progression and treatment outcomes.

The aim of this study was to identify functional markers of the masticatory system and quantitatively assess them in a group of patients diagnosed with TMJ arthralgia.

The following parameters were chosen to capture complementary dimensions of temporomandibular disorder involvement. Bite force indicates the joint’s load-bearing capacity and associated muscular activity, reflecting joint compression and muscle work. Chewing efficiency and chewing frequency represent functional performance, integrating both mechanical and neuromuscular control mechanisms. The pressure pain threshold assesses muscular pain sensitivity and provides insight into peripheral sensitization processes. Range of motion reflects the mechanical and structural status of the TMJ.

For this goal, the following null hypotheses were tested:

**H01.**  *Maximum bite force, chewing efficiency, chewing frequency, pressure pain threshold of the masticatory muscles, and mandibular range of motion would not differ between symptom-free individuals and patients with TMJ arthralgia.*

**H02.**  *No correlations would exist between demographic, functional and pain variables.*

## 2. Materials and Methods

### 2.1. Recruitment Process

Patients diagnosed with TMJ arthralgia following a consultation at the Orofacial Pain Unit at the Centre for Dental Medicine, University of Zurich, Switzerland, between October 2022 and the end of August 2023 were invited by their treating physician to participate in this study. The recruitment of controls took place between July 2019 and May 2020. Participants were invited to take part through verbal means. The participants were given comprehensive written information and oral explanations regarding the study, and provided written informed consent for participation.

### 2.2. Inclusion Criteria

The patient cohort comprised individuals who satisfied the criteria for TMJ arthralgia according to the DC/TMD classification [7], without concomitant myalgia. The control group included subjects without signs or symptoms of temporomandibular disorders (TMDs) according to the DC/TMDs.

Individuals under 18 years of age, pregnant women, and participants unable to comply with the study protocol because of mental disabilities or insufficient proficiency in the German language were excluded from the study. No additional exclusion criteria related to systemic diseases or prior treatments were applied. The study was approved by the Ethics Committee of the state of Zurich (ID 2019-00584) and was performed in accordance with the Declaration of Helsinki.

### 2.3. Data Collection

The DC/TMD protocol was used to confirm the diagnosis of TMJ arthralgia in the patients and exclude the presence of any TMDs in the control group. The following parameters were measured instrumentally: maximum bite force (MBF), chewing efficiency (CE), chewing frequency (CF), the pressure pain threshold (PPT) on the masticatory muscles, and mandibular range of motion (RoM). All measurements were performed by two calibrated examiners (VV, LD).

For the measurement of MBF, a custom-built device was used. The bite-force device was validated through a series of in vitro experiments that were performed in-house, consisting of repeated stepwise measurements of force from 25 to 500 N, with a material testing device (Zwick/Roell, Ulm, Germany) as gold standard. The measurement of bite force could be achieved with an overall average variability below 8 N against the desired force. During the measurements, the sensing unit was placed on the first molars of the lower dental arch. If the first molar was missing, the second premolar was used for the measurement. A stable positioning of the sensor was ensured by using a buccal stopper placed against the tooth. To ensure safety and hygiene, the sensing unit of the device was covered with a disposable, biocompatible silicon cover layer designed to distribute occlusal forces evenly and enhance patient comfort during measurement.

The subjects were then asked to bite as hard as they could and relax the muscles as soon as the subjective maximum comfortable level was reached. Patients had no feedback on the amount of force produced during the assessment. The measurement was performed three times in a row on both sides. A period of 30 s was left between the loading times to allow the masticatory muscles to recover. The measuring unit remained in place until all three cycles were completed [8]. The CE was measured using a two-coloured chewing gum (Hue-Check Gum^®^, Orophys GmbH, Muri bei Bern, Switzerland), consisting of a pink and a blue component that are pressed together before use. For further details pertaining to the protocol, please refer to [9]. The subjects were instructed to chew as usual, switching sides freely. The examiner counted the chewing cycles silently, with the objective of preventing the patient from setting a tempo and/or exerting an influence on the chewing pace. Following 20 cycles of mastication, the examiner intervened by interrupting the process and extracting the chewing gum from the patient’s oral cavity. The chewing gum was compressed to a thickness of 1 mm and scanned on both sides using a flatbed scanner (HP Inc., Palo Alto, CA, USA) [10]. The electronic colorimetric analysis was performed by means of a custom, open-source, image analysis software programme (Viewgum 1.4, dHAL Software, Kifissia, Greece) and the result was calculated as the Variance of Hue (VOH), depending on the degree of mixture of the two chewing gums.

In the arthralgia group, the CF was measured concurrently with the CE. In contrast, the control group was provided with a commercial chewing gum and instructed to chew in accordance with their habitual routine during ten cycles. CF was determined by recording the spatial position of two markers located on the subject’s nose and chin by a standard video camera (Panasonic HC-X1 Ultra Hd 4k Professional Camcorder, Kadoma, Japan). The motion of the markers was tracked analysed by open-source video analysis tool (Kinovea 2025.1.1, https://www.kinovea.org). A chewing cycle consisted of the time between two consecutives peaks of the vertical trajectory of the chin marker. For both groups, only the first ten chewing cycles of the task were analysed and the CF was obtained as the ratio between the number of cycles and their total duration.

The PPT was determined instrumentally with a digital algometer (AlgoMed, Medoc, Ramat Yishay, Israel). The sensor was pressed against the belly of the masseter muscle and the anterior temporalis muscle. The examination was carried out at a central point on the muscle belly, which was palpated beforehand. The test was repeated three times, with a 30 s break between the repetitions. The participants indicated verbally when their individual pain threshold was reached.

The RoM was measured using a standard metal ruler according to the DC/TMD protocol. Pain-free opening (PFO), maximum unassisted opening (MUO), maximum assisted opening (MAO), as well as protrusion (PRO) and laterotrusion (LAT) values from both sides were collected.

### 2.4. Statistical Analysis

Statistical analysis was performed using IBM SPSS Statistics Version 26 (IBM, Armonk, NY, USA). The highest value of the three repetitions was used for MBF. For the PPT, the lowest value of each of the three repetitions in the temporalis muscle and masseter muscle was used for the analysis. This value was selected for analysis in order to avoid potential overestimation of the threshold due to habituation, delayed reporting, or increased tolerance during repeated measurements. This approach provided a conservative estimate of mechanical pain sensitivity.

In the control group, MBF, PPT, and laterotrusion to the left and right were tested for side differences. Since no significant side differences were observed, the left and right sides were averaged for further analysis. The Shapiro–Wilk test was used to test for normality. Normality was achieved by all variables, except for MBF. The variable was tested with the nonparametric Mann–Whitney and also transformed to its Log10, in order to include it in the multivariate model.

Statistical analysis was performed on all observed variables using a multivariate analysis of covariance (MANCOVA), with age as a covariate to control for its potential confounding effects. The significance level was set at α = 0.05. To determine the effect size, Partial Eta Squared was used and the following cut-off ranges were applied: $\eta_{p}^{2}$ ≤ 0.06 were considered a small effect, 0.06 < $\eta_{p}^{2}$ < 0.14 a medium effect and $\eta_{p}^{2}$ ≥ 0.14 a large effect. The differences in the gender distribution between both groups were examined using Fisher’s exact test and the correlations within the arthralgia group was examined using Pearson’s correlation coefficient.

## 3. Results

In the present study, a total of 13 patients (seven female) with ages ranging from 25 to 75 years and a mean age of 52 years (SD = 17) were examined. All thirteen patients reported unilateral pain, of which nine were on the right side according to DC/TMDs. The control group comprised 19 test subjects (six female), with ages ranging from 21 to 77 years with a mean age of 32 years (SD = 16). The descriptive statistics are displayed in Table 1.

No significant differences were observed in terms of gender distribution between the groups. However, a statistically significant difference was observed between the two groups with regard to age (*p* = 0.001).

The average chewing efficiency measured in the arthralgia group was 0.14 ± 0.08 VOH; no between-group comparison was performed for this variable.

All the other variables were compared between groups while controlling for age. The results of the MANCOVA showed that age significantly affected MBF (*p* = 0.008), whereas it had no influence on the means of the other variables.

**Figure 1:**
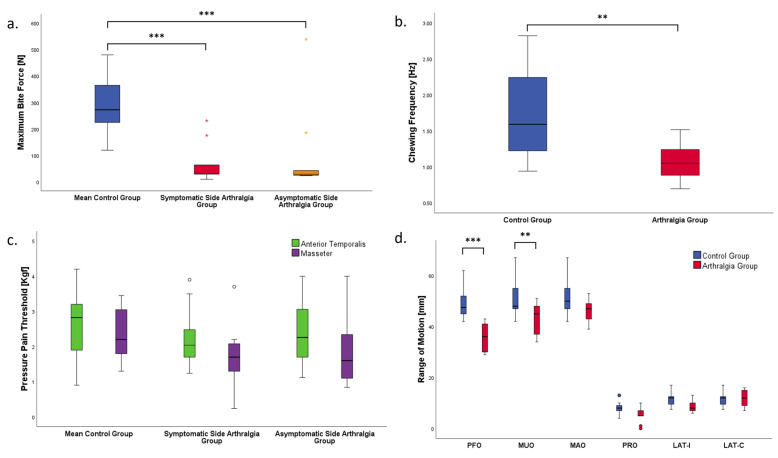
Boxplots representing the group differences for (**a**) bite force, (**b**) chewing frequency, (**c**) pressure pain threshold, and (**d**) range of motion. (°): mild outliers, with values lying between 1.5 and 3.0 times the interquartile range (IQR) from the quartiles (Q1 or Q3). (*): extreme outliers, with values more than 3.0 times the IQR from the quartiles. *p* < 0.01 **, and *p* < 0.001 ***. PFO: pain-free opening; MUO: maximum unassisted opening; MAO: maximum assisted opening; PRO: protrusion; LAT: laterotrusion; I: ipsilateral; C: contralateral.

MBF in the arthralgia group was significantly lower than the values in the control group (*p* < 0.001) (Figure 1a). The effect size was $\eta_{p}^{2}$ = 0.657, which corresponds to a large effect. The non-parametric analysis yielded the same result as the multivariate model.

CF in the arthralgia group was significantly lower than in the control group (*p* = 0.01) (Figure 1b). With a value of $\eta_{p}^{2}$ = 0.29, the effect size corresponded to a large effect. No significant differences in the PPT between the symptomatic and the control group were observed for both masseter and temporalis muscle on either side (Figure 1c).

In the arthralgia group, PFO and MUO were significantly smaller than in the control group (*p* < 0.001, *p* = 0.022 respectively), and there was a large effect size ($\eta_{p}^{2}$ = 0.533, $\eta_{p}^{2}$ = 0.235) (Figure 1d).

Based on these findings, the null hypothesis H01 was partially rejected.

The Pearson’s correlation analysis for the TMJ arthralgia group is presented in Table 2. Age showed a significantly strong negative correlation with MBF on both sides (r = −0.726, *p* = 0.027, r = −0.736, *p* = 0.024 for ipsilateral and contralateral side, respectively). A strong positive correlation was identified for the MBF between the two sides (r = 0.873, *p* = 0.002). A significant strong positive correlation was identified for the PPT of the temporalis muscle on the ipsi- and contralateral side (r = 0.863, *p* < 0.001). The PPT of the temporalis muscle on the side ipsilateral to the pain site correlated positively with the PPT on the ipsilateral (r = 0.595, *p* = 0.032) and contralateral side (r = 0.676, *p* = 0.011) of the masseter muscle. The PPT of the temporalis muscle on the side contralateral to the pain site correlated positively with the PPT on the ipsilateral (r = 0.697, *p* = 0.008) and contralateral side (r = 0.653, *p* = 0.016) of the masseter muscle. The PPT of the masseter muscle of the ipsi- and contralateral side correlated strongly positively with each other (r = 0.829, *p* < 0.001). There was a strong correlation between pain-free opening and the maximum unassisted mouth opening (r = 0.877, *p* < 0.001) as well as the maximum assisted mouth opening (r = 0.857, *p* < 0.001). In addition, the maximum unassisted mouth opening also correlated strongly positively with the maximum assisted mouth opening (r = 0.983, *p* < 0.001). Laterotrusion to the ipsilateral side correlates strongly negatively with the PPT in the temporalis muscle of the ipsilateral (r = −0.612, *p* = 0.026). Based on these results, the null hypothesis H02 was partially rejected.

## 4. Discussion

In this study, a substantial reduction in MBF and CF was observed in patients suffering from TMJ arthralgia when compared to healthy controls. Additionally, significant restrictions in TMJ mobility were identified in the arthralgia group when compared to the control group, as evidenced by pain-free and maximum unassisted mouth opening.

These observations are consistent with the patients’ subjective perceptions, as they frequently complain of reduced chewing force and slower mastication due to pain, muscle soreness and limited range of motion. As demonstrated by Todic et al., patients diagnosed with TMDs exhibited a considerably reduced bite force in comparison to the control group [11]. Furthermore, the authors identified a difference between the genders. The mean bite force recorded for the healthy male population was 939.8 ± 109.7 N, while for the female population it was 743.6 ± 138.0 N. The mean bite force recorded for male patients diagnosed with TMDs was 745.5 ± 129.8 N, and for female patients it was 506.3 ± 174.7 N. In the present study, a substantially lower average value of bite force was observed in subjects of both the symptomatic and asymptomatic groups. Pizolato et al. observed no side differences in symptomatic TMD subjects. However, in accordance with the findings of our own study, a decline in bite force was also identified in patients diagnosed with TMDs in comparison to the control group [12]. On the contrary, Pereira et al. described an opposite trend, in which the mean bite force recorded in the control group was significantly lower than that recorded for the group with TMDs (302 ± 24 N and 326 ± 40 N, *p* < 0.05) [13]. Overall, the substantial variability in the reported absolute values of maximum chewing force between the studies is notable. This heterogeneity may be attributable to differences in measurement methodologies as well as variations in the characteristics of the study populations.

In the present study, the arthralgia group demonstrated a colour-mixing ability of 0.14 VOH, which is higher than the mean value reported by Schimmel et al. in a study evaluating masticatory efficiency in a healthy cohort with average occlusal harmony. In that study, the mean value was 0.254 ± 0.088 VOH (Schimmel et al., 2015) [14].

To our knowledge, this is the first study to quantitatively assess chewing efficiency in patients with TMJ arthralgia. Rodrigues et al. reported similar findings, demonstrating relatively enhanced chewing efficiency in a group of patients with mixed TMDs compared with matched asymptomatic controls [15]. Conversely to the findings of this study, a questionnaire-based clinical study demonstrated a correlation between the subjectively perceived reduction in the chewing ability and TMJ pain and mouth opening limitation [16]. In the present study, CE was evaluated exclusively in the arthralgia group; therefore, no direct statistical comparison could be conducted. Hence, the reference to normative values reported in the literature might provide descriptive context for the observed results. However, such indirect comparisons should be interpreted with caution, as differences in study populations, methodologies, and measurement protocols may influence reported values. In accordance with the findings of this study, an earlier investigation by Schimmel et al. failed to identify a correlation between chewing efficiency and maximum bite force [9]. In comparison with the control group, the arthralgia group exhibited a reduced rate of mastication. This phenomenon was also demonstrated in a study conducted by Brandini et al. [17]. In accordance with the observations recorded, it has been demonstrated by several studies that an increase in the frequency of mastication results in a decrease in mastication efficiency [18]. Thus, a compensatory mechanism may be hypothesised, suggesting that patients suffering from arthralgia chew more slowly in order to prevent pain and maintain chewing efficiency.

It must be noted that the measurement protocols used for CF differed in terms of test material used and number of chewing cycles, which may potentially have affected comparability. For consistency in data evaluation across groups, the analysis was restricted to the first ten chewing cycles of each protocol. This approach ensured that the analysed segments were equivalent in functional phase across both groups, thereby reducing potential bias related to protocol length or fatigue. Furthermore, the device configuration, data acquisition procedures and chewing task itself remained identical for both groups.

No side differences in the PPT were observed for either the masseter or temporalis muscles in patients with unilateral TMJ arthralgia. Additionally, no differences were found between patients with arthralgia and asymptomatic controls. These findings are consistent with the DC/TMD clinical examination, as only patients with arthralgia without concomitant myalgia were included in the study. In contrast to studies reporting a reduced muscular PPT in mixed TMD populations, our findings underscore the importance of diagnostic subgrouping, as inclusion of myogenous TMDs may confound pain sensitivity outcomes [19].

The most significant strength of the study lies in the implementation of well-validated measurement tools, which enabled the quantification of subjectively perceived functional impairments. A limitation of this study is the relatively modest sample size, in addition to the notable age difference between the groups. Moreover, the restricted sample size precluded the differentiation of various potential underlying conditions for the arthralgia, including, for instance, disc displacement or degenerative joint disease.

Methodologically speaking, significant differences in age between the groups were observed. To account for this difference, age was included as covariate in the analysis of variance. Future work should be conducted with age-matched groups recruited within the same time frame to minimise potential cohort effects and reduce residual confounding factors, including inter- and intra-examiner reliability. Additionally, functional data are inherently variable due to extrinsic factors during the experimental session, as well as subject motivation and learning effects, which may further contribute to interindividual differences. Future studies should implement repeated measurements across multiple sessions to enhance reliability.

The results of this pilot study provide a quantitative description of the masticatory system functional markers in patients suffering from TMJ arthralgia. Patients exhibited a reduction in biting force and chewing pace, which is consistent with the complaints frequently reported by patients in everyday clinical practice. Nevertheless, the subjects demonstrated a chewing efficiency comparable to that reported in the literature for healthy subjects. The integration of objective data with the conventional clinical examination facilitates the identification of functional impairments and has the potential to inform longitudinal assessments of therapeutic interventions or disease progression. The instrumental function assessment is hypothesised to be of particular value in cases where verbal communication is restricted due to various factors, including, but not limited to, language barriers or cognitive impairment in the future. The instrumental assessment of masticatory function markers offers valuable additional information for daily clinical practice.

## 5. Conclusions

Patients diagnosed with TMJ arthralgia have been shown to apply reduced bite force and exhibit slower chewing rates in comparison to asymptomatic control groups. The presence of TMJ arthralgia did not appear to compromise mastication efficiency. The instrumental assessment of masticatory function markers provides valuable additional information for daily clinical practice.

## Figures and Tables

**Table 1:** Descriptive statistics for the arthralgia and asymptomatic group.

	Patients		Controls	
	n	Mean (SD)	n	Mean (SD)
***Demographics***				
**Age (y)**	13	52 (17)	19	32 (15)
***Functional performance***				
**Max Bite Force (N)**	9	ipsi: 69.53 (78.56) contra: 48.72 (56.10)		292.24 (100.72)
**Chewing Efficiency (VOH)**	13	0.14 (0.08)		NA
**Chewing Frequency (Hz)**	13	1.13 (0.28)	19	1.58 (0.60)
***Pain sensitivity***				
**PPT Temporalis (kgf)**	13	ipsi: 2.29 (1.08) contra: 2.39 (1.01)	18	2.68 (0.85)
**PPT Masseter (kgf)**	13	ipsi: 1.61 (0.83) contra: 1.76 (0.87)	18	2.29 (0.72)
***Range of Motion***				
**Pain Free Open (mm)**	13	36.77 (7.54)	15	49.53 (6.03)
**Max Unassisted Open (mm)**	13	42.92 (8.38)	15	51.07 (6.51)
**Max Assisted Open (mm)**	13	46.38 (6.59)	15	51.53 (6.35)
**Protrusion (mm)**	13	4.77 (2.77)	15	8.07 (2.49)
**Laterotrusion (mm)**	13	ipsi: 8.92 (2.90) contra: 10.23 (3.98)	15	11.57 (2.60)

**Table 2:** Pearson Correlation Coefficient (r) for the symptomatic (TMJ arthralgia) group, n = 13 except MBF n = 9.

	Age	MBF-I	MBF-C	CE	CF	PPT-T-I	PPT-T-C	PPT-M-I	PPT-M-C	PFO	MUO	MAO	PRO	LAT-I	LAT-C
**Age**	--														
**MBF-I**	−0.726 *	--													
**MBF-C**	−0.736 *	0.873 **	--												
**CE**	0.005	0.114	−0.153	--											
**CF**	0.101	−0.080	0.001	−0.077	--										
**PPT-T-I**	0.345	0.142	0.252	−0.108	0.214	--									
**PPT-T-C**	0.309	0.357	0.387	0.038	0.205	0.863 **	--								
**PPT-M-I**	−0.085	0.440	0.558	0.140	−0.240	0.595 *	0.697 **	--							
**PPT-M-C**	−0.043	0.279	0.507	−0.252	−0.184	0.676 *	0.653 *	0.829**	--						
**PFO**	−0.008	−0.540	−0.276	0.185	0.438	0.111	0.154	0.068	0.094	--					
**MUO**	−0.136	−0.297	0.007	0.188	0.290	0.071	0.225	0.205	0.247	0.877 **	--				
**MAO**	−0.205	−0.273	0.021	0.127	0.327	0.108	0.228	0.210	0.251	0.857 **	0.983 **	--			
**PRO**	−0.488	−0.001	0.190	−0.126	−0.423	0.085	−0.220	0.272	0.410	0.121	0.071	0.119	--		
**LAT-I**	−0.102	−0.372	−0.431	0.278	−0.415	−0.612 *	−0.612 *	−0.306	−0.229	−0.069	0.048	−0.007	0.184	--	
**LAT-C**	−0.028	−0.307	0.110	−0.389	−0.298	0.199	0.204	0.409	0.504	0.429	0.433	0.435	0.435	−0.1354	--

*. Correlation is significant at the 0.05 level (2-tailed). **. Correlation is significant at the 0.01 level (2-tailed). MBF: maximum bite force; CE: chewing efficiency; CF: chewing frequency; PPT: pressure pain threshold; T: temporalis muscle; M: masseter muscle; I: ipsilateral; C: contralateral; PRO: protrusion; LAT: laterotrusion; PFO: pain-free opening; MUO: maximum unassisted opening; MAO: maximum assisted opening.

## Data Availability

The data that support the findings of this study are available from the corresponding author upon reasonable request.

## Abbreviations

The following abbreviations are used in this manuscript:

TMJ	Temporomandibular Joint
DC/TMDs	Diagnostic Criteria for Temporomandibular Disorders
MBF	Maximum Bite Force
CE	Chewing Efficiency
CF	Chewing Frequency
PPT	Pressure Pain Threshold
VOH	Variance of Hue
